# Development of a Nomogram‐Based Online Calculator for Predicting Cancer‐Specific Survival in Patients With Digestive Tract Mixed Neuroendocrine‐Non‐Neuroendocrine Neoplasms (MiNENs): An Analysis of the SEER Database

**DOI:** 10.1002/cnr2.70156

**Published:** 2025-02-19

**Authors:** Jing Tang, Siqi Wei, Guobin Tang, Ping Zhao

**Affiliations:** ^1^ Department of Gastroenterology Guangyuan Central Hospital Guangyuan China

**Keywords:** cancer‐specific survival, digestive tract, mixed neuroendocrine‐non‐neuroendocrine neoplasms, nomogram, online calculator, SEER database

## Abstract

**Aims:**

Mixed neuroendocrine‐non‐neuroendocrine neoplasms (MiNENs) represent a rare and heterogeneous subgroup of neoplasms that typically consist of a neuroendocrine (NE) component, most commonly neuroendocrine carcinoma (NEC), alongside a non‐neuroendocrine (non‐NE) component. They commonly occur in the digestive tract, and their prognosis is influenced by multiple factors. This article aimed to identify factors that affect the cancer‐specific survival (CSS) of MiNENs and develop an effective nomogram‐based online calculator to validate its effectiveness.

**Methods:**

The clinical, pathological, epidemiological, and survival data of patients with digestive tract MiNENs were collected from the Surveillance, Epidemiology, and End Results (SEER) database spanning from 2000 to 2020. Then, the dataset was divided into a training cohort and a validation cohort. The χ^2^ test or Fisher's exact test was utilized to assess differences in demographic and clinicopathological characteristics between the two groups. Kaplan–Meier survival curves and log‐rank tests were employed to conduct survival analysis. Additionally, univariate and multivariate Cox regression analyses were performed to identify potential prognostic factors and develop nomograms and an online calculator for predicting CSS at 1, 3, and 5 years. Lastly, the predictive ability of the online calculator was subsequently compared with the sixth edition of the American Joint Committee on Cancer (AJCC) TNM staging system using the Harrell concordance index (C‐index), the area under the receiver operating characteristic curve (AUC), calibration curve, and decision curve analysis (DCA).

**Results:**

A total of 330 patients were randomly assigned to two groups, namely, the training cohort (*n* = 231) and the validation cohort (*n* = 99). The log‐rank test revealed a significant association between the lower cumulative survival and age ≥ 65 years, poor tumor grade, lack of surgical treatment, TNM stages III and IV, and distant metastasis. In the training cohort, a nomogram incorporating grade, surgery, TNM stage, and tumor metastasis was developed, which demonstrated favorable calibration and discriminatory capabilities. Compared to TNM staging, the nomogram exhibited satisfactory performance in predicting 1‐year, 3‐year, and 5‐year CSS rates. The C‐index value was 0.787 in the training cohort and 0.738 in the validation cohort, respectively. In the training cohort, the nomogram achieved an AUC of 85.81%, 85.86%, and 87.32% for 1‐year CSS, 3‐year CSS, and 5‐year CSS, respectively. In contrast, these AUC values were 78.46%, 81.50%, and 83.88% in the validation cohort, respectively.

**Conclusions:**

The developed online calculator offers a novel approach to predicting the prognosis of patients with digestive tract MiNENs. Indeed, it can accurately predict the CSS of these patients over 1, 3, and 5 years, thereby assisting in enhancing prognosis and formulating appropriate treatment strategies.

## 1 Introduction

MiNENs are a rare class of diseases that can occur in various parts of the body. However, they are more commonly observed in the digestive tract compared to other organs, suggesting a preference for this anatomical site [1]. Notably, they are characterized by the coexistence of both NE and non‐NE histological components and thus display varying morphological characteristics and levels of differentiation. MiNENs, previously referred to as mixed adeno‐neuroendocrine carcinomas (MANECs), were reclassified by the World Health Organization (WHO) in 2017. These tumors consist of both NE and non‐NE components, with each component comprising at least 30% of the tumor composition [2, 3]. According to the 2019 updated classification, tumors can be divided into two components: the NE component and the non‐NE component. The latter comprises adenomas, adenocarcinomas, squamous cell carcinomas, and acinar cell carcinomas. On the other hand, the former consists of well‐differentiated and poorly differentiated neuroendocrine neoplasms (NENs) [4, 5]. However, in the 2022 WHO Classification of Endocrine and NENs, the diagnosis of MiNEN in nondigestive systems is based on the identification of two morphologically distinct components. The 30% threshold has been tentatively retained for digestive MiNEN, awaiting systematic studies to establish its prognostic relevance [6]. Several case reports have established that poorly differentiated NE components and non‐NE components, even if they constitute less than 5% of the tumor volume, can significantly impact patient prognosis [7, 8].

Previous studies have frequently been limited by small sample sizes and have relied on case reports that lack comprehensive analytical data [9, 10]. And, the classification of diseases has undergone revisions, described by multiple terms, reflecting its rarity and heterogeneity [11]. These challenges have resulted in a significant paucity of epidemiological and biological evidence regarding digestive tract MiNENs, including an absence of prognostic information. In turn, this scarcity of valid data has contributed to a lack of consensus and confusion in clinical management [2]. Earlier studies investigating the prognosis of digestive tract MiNENs did not comprehensively include analytical factors, resulting in a lack of consistent or contradictory results. Therefore, there is an urgent need for personalized, predictive models for patients with digestive tract MiNENs. Nomograms provide a reliable solution for integrated biological and clinical models by incorporating various prognostic and determinant variables to yield individualized numerical probabilities of clinical events. In addition, they facilitate rapid calculations via a user‐friendly digital interface. Compared to traditional staging methods, nomograms offer greater accuracy and provide a more comprehensible prognosis [12]. Consequently, they have garnered widespread acceptance in clinical oncology as a reliable and convenient prognostic tool for predicting specific outcomes [13, 14]. The present study utilized the SEER database to identify prognostic clinicopathological indicators of CSS in patients with digestive tract MiNENs and construct a novel nomogram‐based online calculator to serve as an effective prognostic model for digestive tract MiNEN CSS, enabling a more accurate determination of prognosis and assessment of risk factors. By applying this scoring system, clinicians can make more informed clinical decisions. Furthermore, the prognostic ability of this model was compared with that of the 6th AJCC TNM staging.

## 2 Methods

### 2.1 Study Data

**FIGURE 1:**
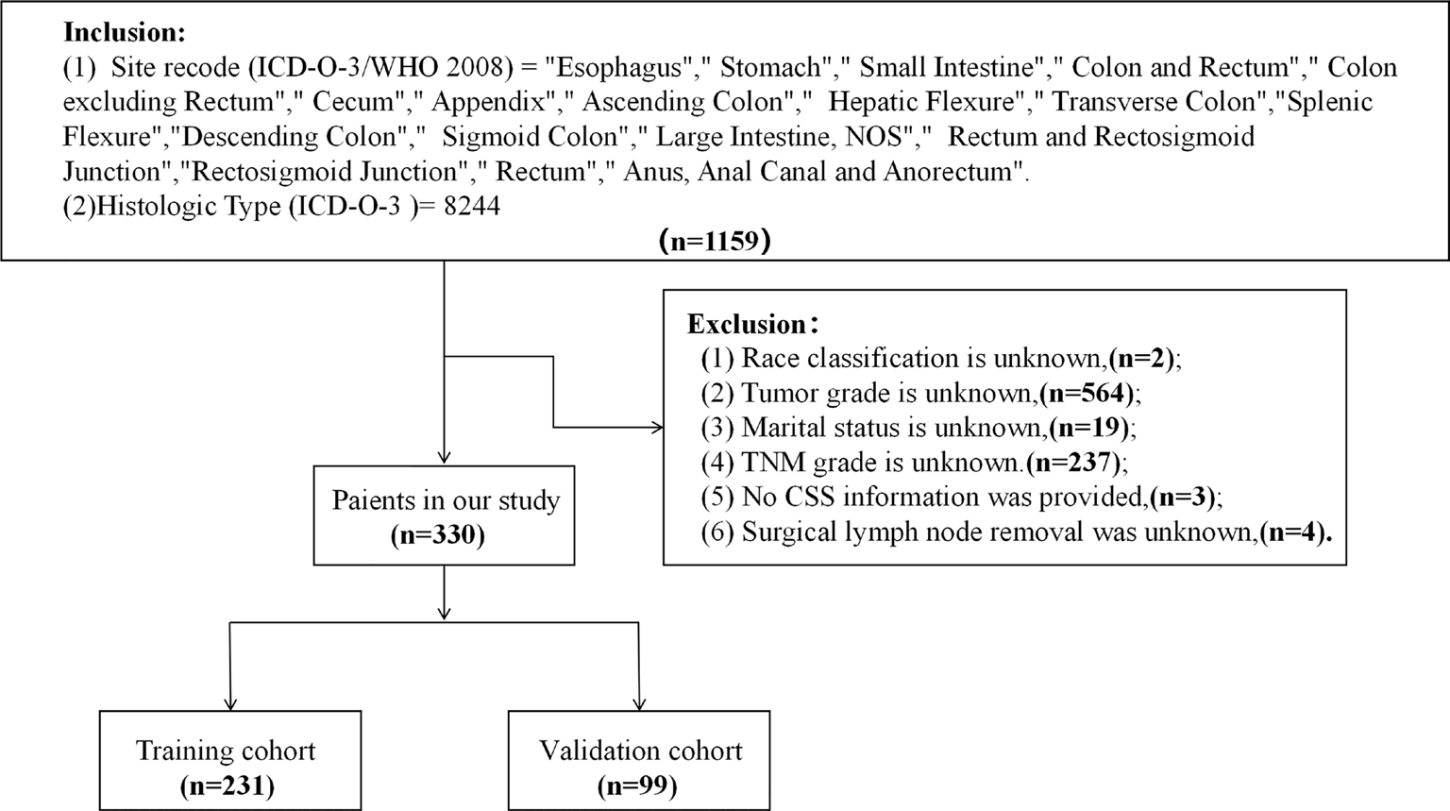
Flowchart of patient enrollment.

Patient data were sourced from the SEER database (Version: 8.4.2; https://seer.cancer.gov/data‐software/), which collects data on patients diagnosed with digestive tract MiNENs between 2000 and 2020. The primary sites of the digestive tract in this study included the esophagus, stomach, small intestine, large intestine, and anal canal. The histology code used for identification was “8244” (ICD‐0‐3) [4, 15]. Patient personal information from the SEER database was not accessible. This study assessed the association between CSS and age, gender, ethnicity, marital status, primary site, tumor grade, surgical intervention, number of lymph nodes excised, number of primary lesions, TNM stage (sixth edition of the AJCC system), and distant metastasis. A total of 1159 patients were initially identified, among which 859 patients were excluded from the study due to the absence of critical information, including ethnicity, marital status, TNM stage, and other relevant factors. Eventually, 330 patients were included in this study and were randomly assigned to a training cohort (*n* = 231) and a validation cohort (*n* = 99) in a 7:3 ratio (Figure 1). The primary outcome measure in this study was CSS, defined as the time from the diagnosis of digestive tract MiNENs to death specifically attributable to digestive tract MiNENs.

### 2.2 Nomogram Construction and Verification

Univariate log‐rank test and multivariate Cox regression analyses were conducted to identify potential prognostic factors. Using the rms package in R software (Version 4.3.2, https://www.r‐project.org/) in the RStudio environment (Version 2023.06.1 + 524, https://rstudio.com/products/rstudio/), a nomogram was developed based on the independent factors. The training cohort was used to construct a nomogram incorporating all significant independent factors for predicting survival. The nomogram was then validated in both the training and validation cohorts. The predictive ability of the nomogram was evaluated using the C‐index and the area under the receiver operating characteristic (ROC) curve (AUC) using the timeROC package, calibration curve, and DCA. An online calculator for public use was generated through shinyapps.io (https://www.shinyapps.io/) using RStudio.

### 2.3 Statistical Analysis

The χ^2^ test or Fisher's exact test was utilized to assess differences in demographic and clinicopathological characteristics between the training cohort and the validation cohort. To identify potential factors influencing CSS, Kaplan–Meier survival curves and log‐rank tests were employed for analysis. Cox proportional hazard models were utilized to estimate hazard ratios (HR, where an observed HR < 1 was associated with a worse prognosis.) All p‐values were two‐sided, and those below 0.05 were considered statistically significant. Data from the SEER database were acquired using the SEER Stat V.8.4.2, and statistical analyses were carried out using the SPSS V.23.0. Kaplan–Meier survival curves were plotted using Prism 8 (GraphPad Software, San Diego, CA, USA) software.

## 3 Results

### 3.1 Demographics and Clinicopathological Characteristics

**FIGURE 2:**
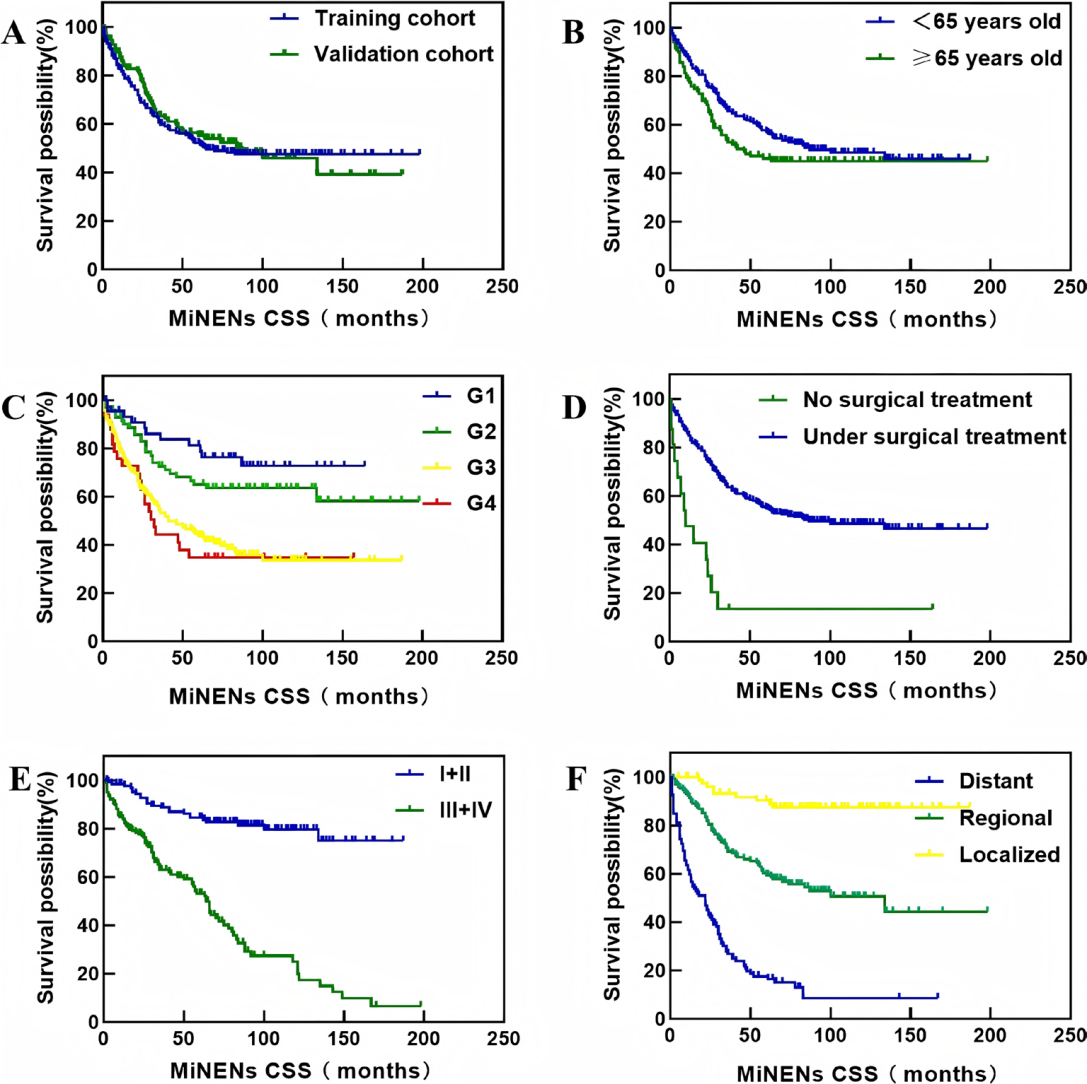
Kaplan–Meier curves and log‐rank test for CSS in patients with digestive tract MiNENs. (A) Training cohort vs. validation cohort; (B) age < 65 years vs. age ≥ 65 years; (C) histological grade G1 vs. G2 vs. G3 vs. G4;(D) no surgical treatment vs. surgical treatment; (E) TNM stage: I + II vs. III + IV; (F) summary stage: distant vs. regional vs. localized.

A total of 330 eligible cases from the SEER database were included in this study and randomly assigned to the training cohort (*n* = 231) and validation cohort (*n* = 99) in a 7:3 ratio. The demographic and clinicopathological characteristics were comparable between the training and validation cohorts (Table 1). The estimated mean CSS of the 231 patients with digestive tract MiNENs was 55.1 months (range 0–198 months) in the training cohort and 56.04 months (range 0–187 months) in the validation cohort. Survival analysis demonstrated no significant differences in CSS between the two groups (Figure 2A, *p* = 0.635).

**TABLE 1:** Demographics and clinicopathological characteristics of the training cohort and validation cohort.

Variables	Training cohort (*n* = 231)	Validation cohort (*n* = 99)	*p*
Age			
< 65 years	146	54	0.140^a^
≥ 65 years	85	45	
Sex			
Male	121	45	0.249^a^
Female	110	54	
Ethnicity			
White	192	81	0.775^a^
Not white	39	18	
Primary site			
Esophagus	2	1	0.484^a^
Stomach	13	7	
Small intestine	8	7	
Colon and anal canal	208	84	
Histological grade			
G1	34	13	0.976^a^
G2	50	23	
G3	122	52	
G4	25	11	
Surgery			
No surgical treatment	16	2	0.072^a^
Under surgical treatment	215	97	
Surgical removal of lymph nodes			
None	52	14	0.093^b^
One to three regional lymph nodes removed	10	2	
Four or more regional lymph nodes removed	169	83	
Number of primary site			
One primary only	176	82	0.181^a^
More than one primary only	55	17	
Marital status at diagnosis			
Not married	102	35	0.145^a^
Married	129	64	
6th AJCC TNM stage			
I + II	88	45	0.223^a^
III + IV	143	54	
Summary stage			
Distant	76	34	0.854^a^
Regional	97	43	
Localized	58	22	

Abbreviation: 6th AJCC TNM stage, TNM stage from the sixth edition of the AJCC system.

^a^ Chi‐square test.

^b^ Fisher's exact test.

### 3.2 Associations Between Variables and CSS of Digestive Tract MiNEN Patients

The log‐rank test revealed a significant correlation between patients aged ≥ 65 years, worse tumor grade, lack of surgical treatment, TNM stages III and IV, distant metastasis, and lower cumulative survival rate (Figure 2B–F, *p* < 0.05). However, factors such as gender, ethnicity, primary location, number of excised lymph nodes, number of primary lesions, and marital status were not significantly correlated with the survival rate (*p* > 0.05) (Table 2).

**TABLE 2:** Univariate and multivariate Cox regression analyses of digestive tract MiNENs CSS in the training cohort.

Variables	Univariate analyses		Multivariate analyzses	
	HR (95% CI)	*p*	HR (95% CI)	*p*
Age				
< 65 years	Reference			
≥ 65 years	0.839 (0.576–1.222)	0.360		
Sex				
Female	Reference			
Male	1.174 (0.817–1.687)	0.387		
Ethnicity				
White	Reference			
Not white	1.372 (0.810–2.326)	0.240		
Primary site				
Esophagus	Reference			
Stomach	1.032 (0.144–7.397)	0.975		
Small intestine	0.972 (0.427–2.213)	0.946		
Colon and anal canal	1.401 (0.571–3.436)	0.462		
Histological grade				
G1	Reference		Reference	
G2	0.237 (0.101–0.554)	0.001	0.293 (0.123–0.693)	0.005
G3	0.299 (0.148–0.606)	0.001	0.350 (0.170–0.721)	0.004
G4	0.928 (0.542–1.590)	0.786	0.655 (0.377–1.137)	0.133
Surgery				
No surgical treatment	Reference		Reference	
Under surgical treatment	3.907 (2.173–7.024)	< 0.001	3.512 (1.880–6.559)	< 0.001
Surgical removal of lymph nodes				
None	Reference			
One to three regional lymph nodes removed	1.147 (0.739–1.779)	0.541		
Four or more regional lymph nodes removed	1.265 (0.513–3.118)	0.609		
Number of primary site				
One primary only	Reference			
More than one primary only	1.278 (0.816–2.002)	0.285		
Marital status at diagnosis				
Not married	Reference			
Married	1.025 (0.710–1.478)	0.896		
6th AJCC TNM stage				
I + II	Reference		Reference	
III + IV	0.127 (0.073–0.223)	< 0.001	0.362 (0.166–0.789)	0.011
Summary stage				
Distant	Reference		Reference	
Regional	20.718 (8.889–48.290)	< 0.001	5.994 (1.893–18.977)	0.002
Localized	6.100 (2.603–14.295)	< 0.001	2.700 (0.929–7.848)	0.068

Abbreviations: CI, confidence interval; CSS, cancer‐specific survival; HR, hazard ratio; 6th AJCC TNM stage: TNM stage from the sixth edition of the AJCC system.

### 3.3 Multivariate Cox Regression Analyses of Digestive Tract MiNEN CSS in the Training Cohort

Multivariate COX regression analysis revealed independent associations between tumor grade (G2: HR = 0.293, *p* = 0.005; G3: HR = 0.350, *p* = 0.004; G4: HR = 0.655, *p* = 0.133), surgical treatment (under surgical treatment: HR = 3.512, *p* < 0.001), TNM stage (III + IV: HR = 0.362, *p* = 0.011), and tumor metastasis (Regional: HR = 5.994, *p* = 0.002; localized: HR = 2.700, *p* = 0.068) with the CSS of patients with digestive tract MiNENs. Conversely, variables such as gender, age, ethnicity, primary tumor location, number of surgically resected lymph nodes, number of primary tumors, and marital status were not significantly correlated with the CSS of patients with digestive tract MiNENs (Table 2).

### 3.4 Nomogram Construction

**FIGURE 3:**
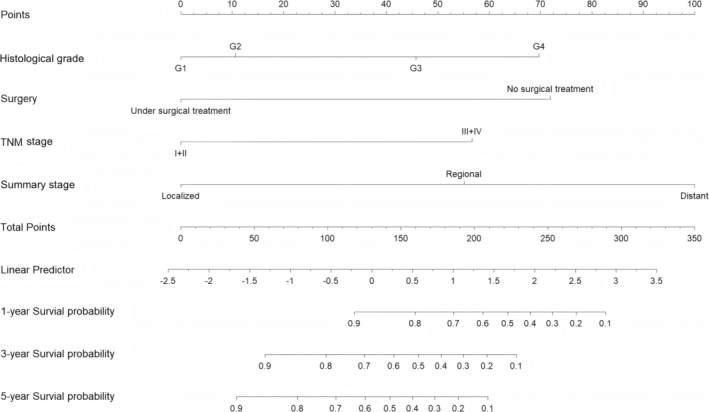
Nomogram for predicting 1‐year, 3‐year, and 5‐year CSS in patients with digestive tract MiNENs, utilizing four factors, namely, histological grade, surgery, summary stage, and 6th AJCC TNM stage.

In this study, nomograms were constructed to predict 1‐year, 3‐year, and 5‐year CSS by incorporating four variables: tumor grade, surgery, TNM stage, and tumor metastasis (Figure 3). As illustrated in the nomogram, metastasis was identified as the most significant predictor of the CSS of patients with digestive tract MiNENs, followed by surgery, tumor grade, and TNM stage. The cumulative values associated with each patient's indicators can be used to predict the patient's 1‐year, 3‐year, and 5‐year CSS values.

### 3.5 Validation and Calibration of the Nomogram

**FIGURE 4:**
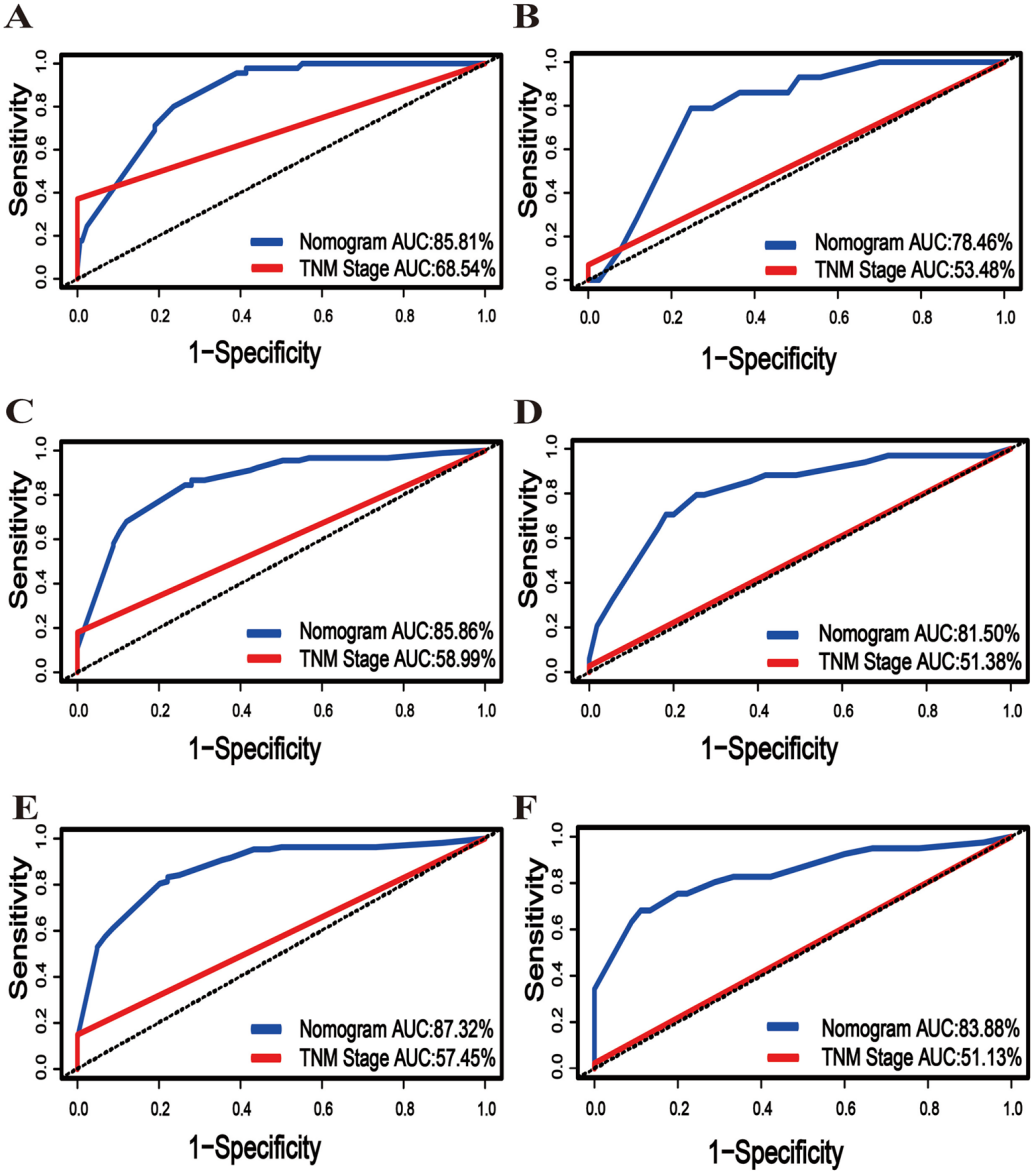
ROC curve of the training and validation cohorts for predicting the 1‐year, 3‐year, and 5‐year CSS of patients with digestive tract MiNENs compared with the 6th AJCC TNM stage. (A, C, E) ROC curve of the training cohort compared with the 6th AJCC TNM stage for predicting 1‐year, 3‐year, and 5‐year CSS; (B, D, F) ROC curve of the training cohort compared with the 6th AJCC TNM stage for predicting 1‐year, 3‐year, and 5‐year CSS.

The C‐index value was 0.787 in the training group and 0.738 in the validation group. Meanwhile, the area under the ROC curve for predicting 1‐year, 3‐year, and 5‐year CSS was 0.858, 0.859, and 0.873 in the training cohort, respectively. More importantly, these values were significantly higher compared to the values of 0.685, 0.590, and 0.575 obtained using the sixth edition of the AJCC TNM staging system. Similar results were observed in the validation cohort, indicating that the area under the ROC curve for predicting 1‐year, 3‐year, and 5‐year CSS was 0.785, 0.815, and 0.839, respectively, which were significantly higher compared to the values of 0.535, 0.514, and 0.511 derived using the sixth edition of the AJCC TNM staging system. (Figure 4A–F).

**FIGURE 5:**
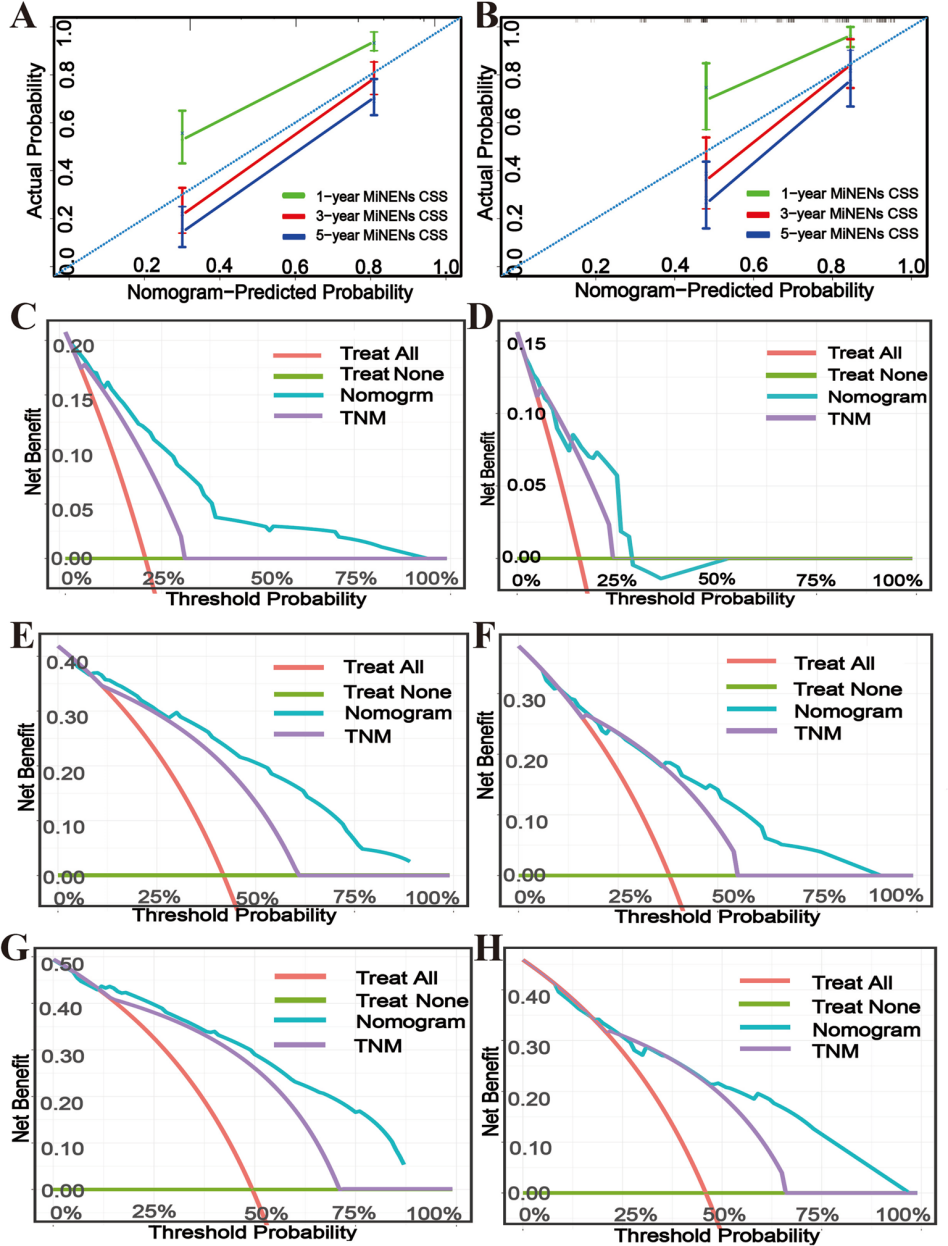
Calibration plots and decision curve analysis for predicting CSS in the training and validation cohorts at 1, 3, and 5 years. Calibration plots of the nomogram in the training (A) and validation (B) cohorts at 1, 3, and 5 years. Decision curve analysis for the nomogram and the 6th AJCC TNM stage in predicting the prognosis in the training (C, 1‐year; E, 3‐year; G, 5‐year) and validation (D, 1‐year; F, 3‐year; H, 5‐year) cohorts.

As anticipated, the calibration curve of the nomogram displayed that the predicted results for digestive tract MiNEN CSS in 1, 3, and 5 years closely matched the actual observed results in both the training and validation cohorts. Particularly, the predicted results for digestive tract MiNEN CSS at 3 years were in close agreement with the actual observed results (Figure 5A,B). At the same time, the DCA curve depicted that the nomogram had higher clinical utility and benefits compared to the TNM staging of the sixth edition of the AJCC TNM staging system for predicting the CSS of patients with digestive tract MiNENs at 1, 3, and 5 years in both the training and validation cohorts (Figure 5C–H). Subsequently, an online calculator was developed based on this nomogram and is available for free use by the public (https://asd330366353.shinyapps.io/DynNomapp/).

## 4 Discussion

NENs represent a distinct category of malignant neoplasms. Epidemiological data indicate that their annual incidence is approximately 5.6 per 100 000 individuals. Tumors that originate from the digestive system are classified as gastro‐entero‐pancreatic neuroendocrine neoplasms (GEP‐NENs), which account for 62%–67% of the total incidence of NENs. Of note, GEP‐NENs are recognized as the second‐most prevalent type of cancer affecting the digestive system, with their incidence rate steadily increasing annually [16, 17, 18, 19]. Given that MiNENs were not classified as a distinct tumor type by the WHO until over a decade ago, our understanding of these tumors remains limited, and there is a lack of in‐depth research [2]. As a result, our understanding of their clinical characteristics remains elusive, with significant controversy surrounding their treatment and prognostic factors. Considering that MiNENs include tumor NE and non‐NE tissue components, their clinicopathological manifestations differ from NENs. Historically, specific data on the prognostic parameters of digestive tract MiNENs were limited, with stage IV being the only relevant parameter for identifying aggressive disease [20]. While surgical intervention is considered the only approach to improve prognosis, its role may be limited, and there is a lack of clinical guidance [21]. In addition to the aforementioned factors, various other elements may influence the prognosis of NENs, encompassing metabolic disorders, the use of lipid‐lowering medications, gender, variations in distant metastatic sites, and the anatomical location of the primary tumor. Elucidating these parameters can assist in personalizing the management of NENs, including tailoring treatment options and identifying potential therapeutic targets. However, it is worthwhile emphasizing that the majority of studies do not specifically address MiNENs within the digestive tract. Furthermore, numerous studies are observational in nature and involve limited sample sizes, which diminishes their overall generalizability [22, 23, 24, 25, 26]. To accurately and objectively obtain survival data for patients with digestive tract MiNENs and to develop highly visualized and user‐friendly predictive tools, this approach can assist in more effectively evaluating patient prognosis and selecting appropriate treatments. As the largest cancer registry, the SEER database encompasses approximately 35% of the U.S. population and serves as a robust resource for conducting population‐based retrospective studies. Consequently, this study opted to utilize the extensive population‐based data from the SEER database in conjunction with the visualization capabilities of nomograms to optimize the prognostic evaluations of MiNENs in the digestive tract.

Herein, 330 patients with digestive tract MiNENs from the SEER database were assigned to two cohorts in a 7:3 ratio. Next, a valid nomogram that included four independent prognostic factors, namely, grade, surgery, TNM stage, and metastasis, was developed. Of note, these factors align with findings from previous studies, reinforcing their positive influence on the prognosis of patients with digestive tract MiNENs. The nomogram, derived from the Cox regression model, was subsequently used to predict 1‐year, 3‐year, and 5‐year CSS in patients with digestive tract MiNENs, and the results highlighted its outstanding discriminatory ability. More importantly, the nomogram exhibited superior predictive power for CSS compared to the sixth edition of the AJCC TNM staging. Specifically, the AUC values for 1‐year, 3‐year, and 5‐year CSS were 0.858, 0.859, and 0.873 in the training cohort compared to 0.685, 0.590, and 0.575 for the AJCC TNM staging system, respectively. Similarly, the AUC values for 1‐year, 3‐year, and 5‐year CSS were 0.785, 0.815, and 0.839 in the validation cohort and 0.535, 0.514, and 0.511 in the AJCC TNM staging system, respectively.

As is well documented, distant metastasis is an established risk factor for poor prognosis in most malignancies [27, 28]. There are three modes of distant spread of MiNENs, with the most common being the transfer of a single NE component, the least common being the transfer of a single non‐NE component, and the transfer numbers of NE and non‐NE mixed components falling between. This may be ascribed to NE components being generally poorly differentiated and dominant. Besides, the absence of locoregional lymph node metastasis is associated with longer recurrence‐free survival (RFS) (*p* = 0.015) [29, 30]. A retrospective study conducted in China enrolling 46 patients diagnosed with digestive tract MiNENs reported that 27 patients died of tumor‐related causes, with a mean survival time of (28.60 ± 15.14) months and a median survival time of 30 months (ranging between 12 and 43 months). Moreover, the study identified distant metastasis (HR = 0.141, *p* = 0.004) as a significant factor affecting patient prognosis [31], in line with the results of this study. Herein, the average survival time of patients with digestive tract MiNENs with distant metastasis was 25.92 months, while the median survival time was 15.10 months. These figures were significantly worse than those of patients with local metastasis, whose average and median survival times were 69.50 months and 70.12 months, respectively. Song Huixin analyzed the treatment data of 767 patients in the SEER database, and their results were consistent with the findings of this study [1]. According to various studies, digestive tract MiNENs are categorized as tumors with an abysmal prognosis. Surgical resection has been identified as an effective approach to extend the survival rate of patients with distant metastasis [32]. Additionally, adjuvant chemotherapy or other treatments may yield beneficial outcomes [2, 33].

Earlier studies have unveiled that surgical treatment can improve the prognosis of patients with MiNENs. In a study conducted by Hans‐Christian Pommergaard et al., surgical resection significantly enhanced patient prognosis, even for patients with stage IV primary tumors with high malignancy, [32] in agreement with the results of this study, wherein CSS was significantly longer in patients undergoing surgical treatment (HR = 3.512, *p* < 0.001) compared to digestive tract MiNEN patients who did not undergo surgical treatment. However, the significant role of surgical resection in enhancing the long‐term prognosis of patients with digestive tract MiNENs is not consistently supported by all studies [21]. Current studies have limitations, including limited sample sizes, inconsistent surgical indications, and a predominant reliance on observational studies [34]. Consequently, a comprehensive evaluation is essential. The behavior of tumors is influenced by their histological grade and degree of differentiation. In other words, tumors with higher histological grades and poorer differentiation tend to develop local or distant metastasis earlier, resulting in a worse prognosis [35, 36, 37]. Tumor pathogenesis is inherently multifactorial. NENs and MiNENs, primarily located in the digestive tract, exhibit significant cellular diversity, which contributes to the heterogeneity of GEP‐NENs and GEP‐MiNENs. To expand our understanding of their pathogenic mechanisms, it is essential to identify factors contributing to the observed increasing incidence. This knowledge can assist in reducing incidence rates and improving patient prognosis. Various factors, including environmental factors, individual characteristics, and genetic components, play a crucial role. A deeper understanding of pathogenic mechanisms is crucial for enhancing treatment strategies and identifying new therapeutic targets [19]. Xiaoyang Xing et al. recruited patients with elderly appendiceal MiNENs and observed similar results. Interestingly, grade III tumors (HR = 2.555, *p* = 0.022) were associated with a worse prognosis compared to grades I, II, and IV tumors. Similarly, this study uncovered histological grades G2 and G3 that were associated with a poorer prognosis (G2: HR = 0.349, *p* = 0.003; G3: HR = 0.441, *p* = 0.004), potentially attributed to the conventional belief that tumors with high‐grade histological components in MiNENs have a poorer prognosis compared to conventional non‐NE adenocarcinomas. However, this perspective overlooks the influence of non‐NE components on prognosis. It is important to consider the impact of both NE and non‐NE components when assessing the outcome of these tumors [38]. In the context of clinical treatment strategies, it is vital to consider the non‐endocrine components of MiNENs. Employing a two‐pronged approach could potentially enhance patient prognosis.

Herein, the 1‐year, 3‐year, and 5‐year CSS of patients with digestive tract MiNENs were determined to be 76.06%, 54.55%, and 45.16%, respectively, consistent with the results of a study conducted in China that reported a 1‐year survival rate of 86.2% and a 3‐year survival rate of 55.1% for MiNEN patients [39]. However, the study from China was a single‐center study that involved a small sample size, focusing exclusively on MiNENs located in the stomach. In contrast, the present study encompassed a broader population and examined a more comprehensive range of factors. These prognostic factors were then used to create a visual online calculator that had greater validity than the 6th AJCC TNM staging. Gastroenterologists can employ this online tool for predicting the CSS of MiNEN patients.

Nevertheless, this study has several limitations that merit acknowledgment. Firstly, this was a retrospective study without dynamic controls. Secondly, a significant number of patients were excluded during the sample selection process due to missing data, and the SEER database lacked detailed information on immune tissue markers, tumor markers, and tumor genetic and molecular characteristics, which may have introduced selection bias. Thirdly, all data used in this study were obtained from the SEER database and have not been externally validated. Considering these limitations, large‐scale prospective randomized controlled trials are warranted to further investigate this topic.

## 5 Conclusions

Digestive tract MiNENs are a rare type of tumor with a poor prognosis, influenced by various factors. A nomogram was generated by incorporating four independent prognostic factors of CCS, namely, histological grade, surgery, the 6th AJCC TNM stage, and the summary stage. Importantly, the developed model can more accurately predict 1‐year, 3‐year, and 5‐year CSS in patients with digestive tract MiNENs compared to the 6th AJCC TNM stage. Thus, it can effectively assess the risk of cancer‐related death and offer good predictive power and practical value. The results of this study are publicly accessible through an online calculator.

## Author Contributions

Conceptualization: Jing Tang, Siqi Wei. Methodology: Jing Tang, Siqi Wei, Ping Zhao. Data curation, formal analysis, supervision: Jing Tang, Siqi Wei, Ping Zhao, Guobin Tang. Writing – original draft: Jing Tang, Siqi Wei. Writing – review and editing: Jing Tang, Siqi Wei, Ping Zhao, Guobin Tang. Visualization: Jing Tang.

## Consent

No patient identification information was involved in this study.

## Conflicts of Interest

The authors declare no conflicts of interest.

## Data Availability

The data that support the findings of this study are openly available in SEER database at www.seer.cancer.gov.
